# From Farm to Fork: *Streptococcus suis* as a Model for the Development of Novel Phage-Based Biocontrol Agents

**DOI:** 10.3390/v14091996

**Published:** 2022-09-09

**Authors:** Emmanuel Kuffour Osei, Jennifer Mahony, John G. Kenny

**Affiliations:** 1 School of Microbiology, University College Cork, T12 K8AF Cork, Ireland; 2 APC Microbiome Ireland, University College Cork, T12 K8AF Cork, Ireland; 3 Food Bioscience, Teagasc Food Research Centre Moorepark, Fermoy, P61 C996 Cork, Ireland; 4 VistaMilk SFI Research Centre, Fermoy, P61 C996 Cork, Ireland

**Keywords:** phage, prophages, food, zoonosis, *Streptococcus suis*, anti-viral defence

## Abstract

Bacterial infections of livestock threaten the sustainability of agriculture and public health through production losses and contamination of food products. While prophylactic and therapeutic application of antibiotics has been successful in managing such infections, the evolution and spread of antibiotic-resistant strains along the food chain and in the environment necessitates the development of alternative or adjunct preventive and/or therapeutic strategies. Additionally, the growing consumer preference for “greener” antibiotic-free food products has reinforced the need for novel and safer approaches to controlling bacterial infections. The use of bacteriophages (phages), which can target and kill bacteria, are increasingly considered as a suitable measure to reduce bacterial infections and contamination in the food industry. This review primarily elaborates on the recent veterinary applications of phages and discusses their merits and limitations. Furthermore, using *Streptococcus suis* as a model, we describe the prevalence of prophages and the anti-viral defence arsenal in the genome of the pathogen as a means to define the genetic building blocks that are available for the (synthetic) development of phage-based treatments. The data and approach described herein may provide a framework for the development of therapeutics against an array of bacterial pathogens.

## 1. Introduction

The global livestock industry is a major contributor to food security and economic development, with a value of about 1.4 trillion dollars [1]. The Food and Agriculture Organisation (FAO) estimates that the livestock sector accounts for 40% of the overall agricultural output, with about 1.3 billion people depending on the sector for livelihood and food security [2]. However, infectious diseases threaten the sustainability and growth of the industry. Viruses such as porcine reproductive and respiratory syndrome virus (PRRSV) of swine, bovine herpesvirus 1 of cattle, and infectious bronchitis virus (IBV) of poultry cause severe contagious diseases within the livestock industry. Notwithstanding, bacteria are the key aetiological agents implicated in animal microbial diseases. Bacterial infections such as those arising from *Campylobacter* spp., *Salmonella* spp., *E. coli*, *Clostridium* spp., *Listeria monocytogenes* and *Streptococcus suis* take a significant toll on animal welfare and exert enormous financial losses on the industry [3]. It has been estimated that between 2000 and 2010, zoonotic infections caused about $20 billion and $200 billion in direct and indirect losses, respectively [4,5]. In Australia, it was reported that sales losses and disposal of affected pigs had an associated cost of AUD$ 10–30 million. These losses accounted for a 16–37% reduction in the gross income in the affected regions [6].

## 2. Current Control Measures and Alternative Approaches: A One Health Perspective

In the 20th century, the concept of “One Medicine” became a popular topic in public health [7]. This concept recognised the similar paradigm between veterinary and human medicine, particularly in their shared anatomy, physiology and comparative pathologies [8,9]. This idea has been expanded further to include the environment in a new concept known as “One Health”. One Health is a collaborative approach that focuses on the design and implementation of policies, programmes and legal frameworks aimed at achieving better health outcomes [10,11]. Areas of relevance include environmental degradation, food safety and security, zoonoses and antimicrobial resistance. Thus the prevention and control of bacterial infections is essential for not only the sustainability and growth of the industry but the overall health of the ecosystem. On the farm, management practices in nurseries and the introduction of carrier younglings influence the spread of infection among herds [12,13]. Such management practices are almost universal among livestock farms, which makes the control of infection difficult. The complete eradication of infections is unlikely, but informed strategies have been proposed for infection management on farms. Robust biosecurity, therapeutic and vaccine intervention, as well as hygiene are among the measures used to prevent or control infections on farms [14,15].

Vaccination with autogenous bacterins, a suspension of attenuated or killed bacteria isolated from a specific herd and administered to nonimmune animals within the herd/flock, is a standard practice in infectious disease prevention [16]. Although it has been shown to be effective in controlling the incidence of bacterial infections on farms, these autogenous vaccines are mostly practical on a farm-by-farm basis [17,18,19]. This can be attributed to the prevalence of more than one serotype of a bacterium on an affected farm. A universal vaccine that offers cross-protection against multiple serotypes or strains of a serotype remains elusive due to high genetic diversity, such as that observed in streptococcal infections. Research to address this challenge by developing a universal commercialised vaccine is ongoing, with several subunit vaccine candidates already reported [20]. Additionally, technologies exist that allow for the incorporation of mRNA into carrier molecules to improve half-life and facilitate rapid uptake and expression in the cytoplasm [21]. The protective activity of mRNA vaccines against bacteria was first reported in a preclinical mice model of streptococcal infection. The antigens streptolysin O of Group A *Streptococci* (GAS-SLOdm) and pilus 2a backbone protein of Group B *Streptococci* (GBS-BP-2a) were the selected prototypes of bacterial proteins (antigens) [22]. This vaccine approach could explore different bacterial immunogenic protein-encoding transcripts (mRNA) as potential mRNA vaccine candidates, which, if developed, could potentially replace the current moderately effective autogenous vaccination.

The administration of antibiotics is the most widely implemented measure to prevent (prophylaxis), control (metaphylaxis) and treat (therapeutic) bacterial infections. While the other strategies have proven effective, the use of antimicrobials on farms—particularly as a therapeutic measure—remains a widely adopted practice [23]. Tetracyclines, beta-lactams, bacitracin and macrolides are the most commonly used classes of antibiotics for bacterial infections [24,25]. The application of antibiotics in the industry presents a number of global health concerns. Owing to their broad spectrum of action, antibiotics may clear infectious agents along with other important members of the normal mucosal microbiota, thereby opening up an opportunity for the proliferation of opportunistic pathogens, which could lead to secondary infections [26]. Furthermore, the development of resistance against antimicrobials by infectious agents is a major setback to sustainably using such chemotherapeutics as infection control measures. As a result, the World Health Organisation (WHO) in 2019 declared antimicrobial resistance (AMR) as one of humanity’s top ten public health threats [27,28]. The implications of AMR in livestock involve (1) economic losses due to increased mortality caused by antibiotic insensitive strains; (2) the flow of AMR genes (ARG) from resistant strains to other strains and to different bacterial species [29]. AMR is a One Health concern as it is not only limited to the human–livestock interface but to environmental reservoirs such as water bodies, animal manure and soil [30,31,32,33,34]. The global consumption of antibiotics by livestock has currently surpassed usage in humans [29,35]. Measures have been designed and implemented to regulate the utilisation of antimicrobials in the livestock industry. In 2006, the EU banned the use of antibiotics as growth promoters (AGPs) in livestock farming [36]. As of 2019, 70% of the 160 member countries of the World Organisation for Animal Health (OIE) did not use AGP, as reported in their fifth survey on monitoring quantities of antimicrobial usage [37].

### Alternatives to Antibiotics

With recent increased stringency in regulatory frameworks pertaining to antibiotic usage, new prophylactic and therapeutic strategies are required and actively being explored. These include medium chain fatty acids (MCFA) [38,39], lysozymes, natural anti-inflammatory agents [39,40] and bacteriocins [41,42]. Bacteriophage (phage) biocontrol is another alternative strategy that is under consideration as a measure to act against bacterial infections and contamination in food production (and human medicine). This review will focus on the potential of phages as antimicrobial agents, the current trends in the application of phages in the treatment of livestock, and the prospects of phages in the control of *S. suis*.

Bacteriophages or “bacteria eaters” are viruses that infect and kill a cognate bacterial host. These bacterial viruses are ubiquitous and have an estimated abundance of 10^30^ to 10^32^ particles on Earth [43]. Their presence has been reported in a wide array of environments, including waterbodies, sewage, animals and humans as well as extreme environments such as hot springs and waste treatment plants [44]. The genetic material of phages, which may be DNA or RNA, is encapsidated in a protein coat. The tailed phages account for over 96% of all characterised phages, with a structural organisation of a capsid connected to the tail by a collar structure. The life cycle of phages and their early application in the treatment of bacterial infections have been reviewed in detail elsewhere [45,46].

After their discovery, many studies investigated the potential of phages as an antibacterial in clinical cases, often with contradictory treatment efficacies reported. However, some of the challenges encountered in early phage therapy trials have been attributed to knowledge gaps in phage biology, procedural errors in experiments, and inadequate technology to study host–phage interactions [47]. These challenges affected the development and application of phages in Western Europe. Additionally, the discovery and commercialisation of antibiotics in the 1940s resulted in a decline in the therapeutic usage of phages in human infections. Nevertheless, phage research and commercial production progressed in Eastern Europe and later in the USA [45]. The extensive, and often improper, usage of antibiotics has contributed to the emergence of antibiotic-resistant bacteria. Moreover, the diminished investment into antibiotic research by the pharmaceutical industry has drastically affected the discovery and approval of new antibiotics [48]. The alarming prognosis of multidrug resistance and the shortage of on-shelf antibiotic compounds has rejuvenated global phage therapy research efforts.

Phages target bacteria in a highly host-specific manner but do not infect eukaryotic cells and have no reported serious adverse effect on treatment subjects. These desirable properties render some theoretical advantages to phages relative to antibiotics as prohylactic or treatment candidates. Table 1 summarises the attributes of phages as a suitable antibacterial agent and the limitations associated with their usage.

**Figure 1:**
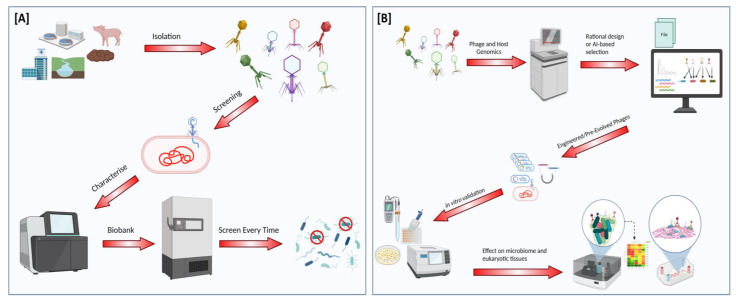
Schematic representation of (**A**) conventional preclinical phage characterisation and (**B**) novel approaches to phage characterisation.

Conventionally, phage application in therapy, biocontrol or decontamination began with phage isolation followed by screening against a panel of host strains. In vitro characterisation such as determining phage titres, pH, thermal stability and growth kinetics was carried out, and phage stocks were prepared and stored for future investigations or application (Figure 1A). Novel approaches to phage research harness high throughput sequencing technology and computer-based analyses to study phage–host interactions (Figure 1B). Data generated from these analyses may subsequently be used in the rational selection of superior candidates for bacterial control.

While the use of phages has not been approved for commercial use in most countries, they are used on a case-by-case basis in treating patients for whom currently approved therapeutics have failed (compassionate phage therapy). Table 2 below summarises some of the recent compassionate uses of phages in human infections. The next subsections explore some recent applications of phages (as single phage preparations or combinations of multiple phages in a “cocktail”) in agriculture and the food industry.

## 3. Phages in Agriculture and Food Industry

The world is currently moving toward “greener” crops and animal food products, with an attitudinal shift in consumer preference for antibiotic-free and sustainably farmed and or processed products [59]. On farms, the restriction of antibiotic use and AMR are indicative that exploring alternative infection control is imminent. Thus, the antibacterial and environmentally friendly properties of phages support their potential use across the agricultural supply chain, such as in the “farm to fork strategy”, which aims to provide safe, sustainable and eco-friendly food systems.

### 3.1. Phages in Primary Food Production

*Salmonella* spp. remains a leading aetiological agent of human food poisoning and gastrointestinal infections in swine, poultry and other livestock. As an enteric pathogen, intestinal contents are usually affected, which predisposes carcasses to contamination during slaughter and packing. To prevent this, controlling colonisation of the pathogen prior to slaughter is practised. Thanki et al. determined the efficacy of a two-phage cocktail in reducing *Salmonella enterica* colonisation in piglets. Weaner meals infused with spray-dried phages were fed to experimentally infected piglets over five days. Phage treatment significantly reduced *Salmonella* carriage in the stomach tissue by 1 × 10^1^ CFU/g, duodenum tissue (1.05 × 10^2^ CFU/g), colon content (1 × 10^1^ CFU/g) and caecum (1 × 10^1^ CFU/g). A 16 S rRNA gene sequencing analysis demonstrated no deleterious effect on the overall microbiome following phage treatment. However, a group that received a phage diet yielded higher abundance of *Prevotellaceae*—a Bacteroidete that has been associated with disease resilience and growth performance in pigs [60]. This study is possibly the first report of scale-up data in a mainstream commercial spray dryer [61]. A similar study reported the clearance of *Salmonella* in the caecal samples (95%) and ileal samples (90%) when pigs were orally administered a microencapsulated phage cocktail post-bacterial challenge [62]. Gebru et al. also demonstrated that anti-*Salmonella* Typhimurium phage in diets significantly reduced bacterial shedding in pig faeces while increasing growth performance [63]. Assessment of phage efficacy in *Salmonella* and other infections associated with poultry has been extensively reviewed by Mosimann et al. [64]. Contrastingly, very few studies have reported the potential of phages in bacterial infections of cattle. Apart from some in vitro and mice model studies, no reports of farm trials were found in a non-exhaustive search using the keywords “phage”, “cattle” and “biocontrol” in Google Scholar, Scopus and PubMed, [65,66,67]. This may be due to the cost associated with enrolling enough cattle in a study that would be suitable for making statistically sound inferences, as well as the space required to house the animals for the period of the study.

Avian pathogenic *Escherichia coli* (APEC) is an extra-intestinal *E. coli* that causes local and systemic infections in avian species such as chicken, turkey and ducks. Some common colibacillosis caused by APEC include egg peritonitis, pericarditis, salpingitis, airsacculitis, cellulitis and osteomyelitis [68]. Collectively, these infections contribute to a reduction in egg production (20%) and mortality of 20–40%, with birds between 4–6 weeks of age being the most susceptible [69]. APEC may be the singular pathogen in an infection or a preceding, concomitant or secondary infection in birds [70,71]. Tawakol et al. evaluated the efficacy of phages in a single APEC infection and a mixed APEC and infectious bronchitis virus (IBV). Challenging birds with APEC and/or IBV resulted in a mortality rate of 16% and 29% by day 7 and day 8, respectively. Conversely, intratracheal administration of phage preparation during the course of the study prevented mortality in birds challenged with APEC and APEC + IBV and decreased IBV and APEC shedding in these two treated groups [72]. A similar observation was made in a more recent study in which chickens were challenged with a multidrug-resistant and strong biofilm producer, *E. coli* O78. Birds that were treated with phages following challenge presented less severe clinical manifestations compared to the challenged untreated group. In addition, administration of phages offered complete protection, whereas in the untreated group, a mortality rate of 26.7% was recorded [73]. The superior efficacy of phage cocktails compared to a single phage preparation was demonstrated in colibacillosis in quails. A reduction in mortality from 26.5% to 13.6% was reported following an intramuscular injection with a single phage and a cocktail, respectively. Both phage-treated groups resulted in better clinical and survival outcomes compared to the challenged untreated quails (46.6% mortality rate) [74].

*Campylobacter* is a zoonotic pathogen responsible for ~25% of all human diarrheal illnesses globally [75]. The bacterium is prevalent in several food animals, particularly in the avian gut, and could contaminate carcasses during postharvest processing. Richards et al. demonstrated that a two-phage cocktail could selectively infect and reduce *C. jejuni* populations in the gut of broiler chickens by 2.4 log CFU/g without disturbing the resident gut microbiome [76]. One major hurdle in the phage application in *C. jejuni* infection is the reported in vitro and in vivo emergence of phage-resistant isolates (up to 13% of isolates) [77]. However, rational selection of phages with diverse genetic characteristics can be used to reduce the challenge. Historically, based on genome size, morphology, and the host receptor employed during infection, *Campylobacter* phages were grouped into I, II and III [77]. In a 31-day trial, administering one group III phage followed by a group II phage significantly decreased *C. jejuni* counts in broiler chickens by 3.0 log units compared to a two-phage cocktail made of only group III phages (1.0 log unit). In addition to the higher reduction, combining group II and III resulted in lower levels of phage-resistant isolates compared to using a single phage or a homogenous phage cocktail [78]. The efficacy of this multi-group *Campylobacter* phage cocktail design has been validated in vitro using several group II and III phages [79]. The application of phage therapy in *C. jejuni* infection in chickens has been reported by other studies with varying degrees of efficacy [80,81,82].

*Clostridium perfringens* Type A strains form part of the resident microbiota of animals; however, toxin-producing strains are implicated in a number of livestock diseases, including necrotic enteritis in poultry, necrohaemorrhagic enteritis in cattle, haemorrhagic diarrhoea in swine, and pigbel in humans. The multi-host nature of this pathogen makes it an ideal target for phage therapy. The phage cocktail “INT-401” was examined for its effectiveness in chicken necrotic enteritis (NE). As part of the study, three methods of oral administration were tested. Throughout the 42 days of observation, all phage treatments significantly (*p* < 0.05) reduced mortalities due to NE to 0–14.0% compared to the mortality rate among the challenged untreated birds (64%) or the antibiotic/medicated group (50%). In-water delivery of phages was the most effective delivery method, with 0% mortality due to NE [83]. In a recent study, 14-day-old broiler chickens were experimentally infected with *C. perfringens* via oral gavage. Phage treatment via similar routes significantly improved clinical parameters and reduced gross lesions compared to the infected untreated birds. The mortality rate was 30% in the phage-treated group compared to the infected untreated group (55%) [84].

Aquaculture is one of the fastest growing industries in agriculture. The FAO reported that fish accounted for 17% of all animal protein consumed globally in 2017 [85]. Aquaculture production reached an all-time high of 84.1 million tonnes in 2018, higher than bovine meat or ovine meat production [86]; however, microbial infections threaten the growth and sustainability of this industry. Phage therapy has been used as a control strategy in bacterial diseases of fishes. In the terrestrial livestock setting, the delivery, diffusion and access of phages to infection sites or surfaces sometimes limit treatment outcomes. However, the liquid medium of aquaculture allows easy access of phages to pathogens in the water (farm environment) and to fishes through the gills [87]. This has been demonstrated in *Aeromonas hydrophila* infection in Nile tilapia. *A. hydrophila* is a zoonotic pathogen reported as the leading cause of septicaemia in freshwater fish [88]. In their study, Dien et al. demonstrated that successive introduction of the inoculum and phage (multiplicity of infection, MOI 1) in water significantly improved fish survival rates (up to 80%) compared to the 25% survival rate among fish in the infected untreated water. Furthermore, phage treatment reduced *A. hydrophila* in fish as well as stimulated the development of specific IgM against the MDR *A. hydrophila* [89]. The immunogenic and immunostimulatory capability of phages in humans and animals have been described by others [90,91,92]. However, an MOI of 100 was required to achieve a similar survival rate (85%) in turbot intraperitoneally infected with *Vibrio harveyi* and administered in-feed phage [93]. The differences in the efficacies of phage treatment between the two studies may be due to the delivery method utilised in the studies. Infusing phages in food pellets may have locked phage particles on food substrate and hence therapeutic phages were available to turbot mostly by feeding ad libitum. By dispensing directly in water, free phages became readily accessible to the Nile tilapia, and thus a comparatively lower MOI was required to produce a similar level of protection recorded in the turbot study. Other studies make reference to MOI when reporting phage dosages. However, factors such as target bacterium, animal species, phage infection kinetics and environmental conditions in the experimental setup could also account for the phage dosage required to offer protection [94]. It is likely that lowering the challenge inoculum by 10-fold would not be resolved by treating with a 10-fold lower phage dose. Thus, reporting of phage dosages could include more descriptive information, such as Poisson distribution and killing titres, which can improve reproducibility and ensure consistency in phage therapy and in the biocontrol literature [95]. Other factors that affect the efficacy of phages include the method of delivery. Phages can be inactivated by the acidic environment of the stomach and enzymatic degradation or neutralised by the immune system, which impacts efficacy of treatment in farm animals [96]. Encapsulating phages in biomaterials such as biopolymers and the use of nano-carriers have been applied for improved stability and accessibility of infection sites and the overall half-life of phages compared to free phages [97,98]. Phages for disease control in aquaculture have been extensively reviewed by Culot et al. and other groups [87,99,100,101].

### 3.2. Other Applications of Phages in Livestock and Food Production

#### 3.2.1. Phages as Growth Promoters

Phages have been demonstrated to play a significant role in growth promotion and improving gut health. Upadhaya et al. [102] assessed the effects of a commercial multi-species phage cocktail on growth performance and other production traits as well as gut health in healthy broiler chickens. A higher body weight gain was observed in birds that received phage-supplemented feed than in birds fed with only the basal diet. The effects of the antibiotic control diet and multi-species phage cocktail supplemented diet on growth performance, barrier function and gut microbiota were described in another study. A key observation was that at 200 mg/kg, there was no significant difference in growth performance compared to the antibiotic diet. Moreover, a 400 mg/kg and 600 mg/kg phage diet increased the daily feed intake, average daily gain and final body weight (*p* < 0.05) and reduced the incidence of diarrhoea in weaned piglets (*p* < 0.001) [103]. Studies that report on the effects of dietary phage supplements in healthy unchallenged food animals are scarce, but many recent studies in phage application in food and animal production include parameters that measure growth performance in their experimental design [61,63,83,104].

#### 3.2.2. Phage Therapy in Crop Production

Phages have also been evaluated as biocontrol agents in crop production. *Pseudomonas syringae*, *Pectobacterium* spp., *Ralstonia solanacearum*, *Dickeya solani* and *Xanthomonas* spp. are some of the leading causes of bacterial diseases in crops. Phages that target these plant pathogens have been isolated and characterised as safer alternatives to conventional broad spectrum chemotherapeutics. Bacterial canker caused by *Pseudomonas syringae* is an economically important disease in farming. The efficacy of single phages in controlling canker was evaluated by spraying the leaves of two-year-old cherry trees with *P. syringae*. Phage treatment almost completely cleared the bacterial population (down to < 10 CFU) by the fifth week [104]. *Dickeya solani* and *Pectobacterium* spp. account for half of all *Enterobacteriaceae* soft rot (SRE) in angiosperms, including food and ornamental crops. A six phage cocktail was used to control soft rot in potato tubers following a 5-day incubation. Washing tubers in a phage preparation significantly reduced (*p* < 0.0001) maceration in *D. solani* infection [105]. Phage treatment significantly reduced disease severity (64%) in tubers and incidence of soft rot (61%) among potato tubers *Pectobacterium atrosepticum* [106].

#### 3.2.3. Phages as Biopreservatives and Biosanitisers

After harvest, both animal and plant products are susceptible to bacterial contamination and spoilage while in storage or undergoing postharvest processing. The FAO estimates show that the global economy loses $220 billion annually due to plant diseases [107]. Moreover, in their latest (2015) global report, the WHO’s Foodborne Disease Burden Epidemiology Reference Group (FERG) reported that over 600 million cases of foodborne illness were implicated in 420,000 deaths [108]. Besides colonising food products, bacteria can attach to materials, including stainless steel (SS), polyethylene, rubber and glass by forming an extracellular polymeric matrix (biofilms). The persistence of biofilms on food contact surfaces presents a major challenge in the food industry by damaging equipment, and contaminating food products, thereby increasing production costs. Thermal and non-thermal treatments are routinely used to remove biofilms from surfaces; however, in addition to their cost, these methods can alter the nutritional and organoleptic profiles of food [109,110]. Moreover, several studies have reported disinfectant-resistant biofilms at various stages of food production [111,112,113]. The antibacterial activity and the natural appeal of phages have been exploited in food processing to extend the shelf life of products, prevent food spoilage and decontaminate surfaces (Table 3). The application of phages in food preservation has been reviewed by others in detail [114,115].

Several phages against ESKAPE (*Enterococcus faecium*, *Staphylococcus aureus*, *Klebsiella pneumoniae*, *Acinetobacter baumannii*, *Pseudomonas aeruginosa* and *Enterobacter* species) bacteria have been characterised and assessed in vivo for their potential in veterinary medicine and the food industry, with several commercial phage preparations (Table 4) already approved for use in food safety [130]. However, there are other animal bacterial pathogens of One Health importance that are poorly explored as targets for phage therapy. One such bacterial agent is *Streptococcus suis*, a major cause of respiratory and systemic infections in swine herds.

## 4. Streptococcus suis and Its Phages: A Case Study

*S. suis* is a gram-positive pathobiont of swine that commonly colonises the upper respiratory tract [131] and, to a lesser extent, the digestive and genital tracts of these animals [132]. However, some *S. suis* strains have evolved to become virulent, and in 1954, the first case of invasive infection was reported among piglets [133,134]. *S. suis* strains have been classified into 35 serotypes (1 to 34 and 1/2) based on the antigenicity of their capsular polysaccharides (CPS) [135]. DNA homology and biochemical profiling of the serotypes have led to the reclassification of serotypes 20, 22 and 26 as *Streptococcus parasuis*, 32 and 34 as *Streptococcus orisratti*, and 33 as *Streptococcus ruminantium* [136]. For diagnostic and surveillance purposes, all 35 serotypes are generally reported as *S. suis*. The prevalence of serotypes varies among different locations; however, most clinical cases worldwide have been associated with serotype 2 [137].

In addition to the CPS, which is a critical virulence factor of *S. suis*, proteins such as suilysin (Sly), DNase, muramidase-release protein (Mrp), surface antigen protein, VirA, and extracellular protein factor (Epf) contribute to the overall pathogenicity of the bacterium [138,139,140,141]. Infection in pigs by pathogenic and opportunistic pathotypes may arise from vertical transmission from sow to piglet during or soon after parturition, or by horizontal transmission such as in nose-to-nose contact among herd members [142]. Colonisation often goes undetected since it is not associated with symptomatic expression of disease. However when established, the bacterium may disseminate systemically, resulting in early manifestation, including fever, inappetence, depression, nystagmus and spontaneous death among infected piglets. Lesions due to polyarthritis, meningitis and endocarditis may also be observed [143].

*S. suis* is the most prevalent bacterium implicated in systemic infections in swine [144]. Regarded as a production disease, reporting of *S. suis* infections is not obligatory in many countries and hence data on global prevalence is limited [145]. This hampers efforts to estimate economic cost and the structuring of standardised control measures within the industry. The incidence of diseases among a given herd is typically lower than 5% and is a function of the extent of the prophylactic application of antibiotics [146]. An evaluation of the economic impact of *S. suis* on Spanish, Dutch and German farms determined that the cost per pig was € 0.60–1.30. Using a stochastic model, the study reported higher morbidities and mortalities in nurseries than in any other production stage. This reflected the higher cost of metaphylactic (treatment of animals with no signs of disease that have been in close contact with other animals with clinical manifestation) measures in piglets compared to that of autogenous vaccines for sows [147]. In 2005, Bennett et al. estimated that losses arising from *S. suis* type II infections alone were £100,000–1.3 million annually in Great Britain [148]. The loss is higher in Vietnam, where $370,000–500,000 went into direct costs and $2.27–2.88 million to indirect costs annually from 2011–2014 [149].

As is the case with many viral and bacterial pathogens, the threat posed by *S. suis* is not limited to the porcine industry [137]. Within 15 years of the first report of *S. suis* infection among piglets, a report of a human infection was identified among meningitis patients in Denmark [134,150]. In the same year, cases of *S. suis* meningitis and septicaemia among patients were recorded in the Netherlands and other European countries, suggesting that the pathogen may have crossed the species barrier long before it was first reported [151]. Recent time-dated phylogenomic studies employing analyses of single-nucleotide polymorphisms (SNPs) in *S. suis* genomes suggest the emergence of human-associated clade from swine isolates originating from western Europe. Using representative samples from six continents, Dong et al. traced the most common recent ancestry of human-associated clade to 1802–1855 [152]. A population expansion of this virulent zoonotic clade in the mid to late 1900s correlates with the extensive implementation of intensive pig rearing—where farmers were in close contact with *suis*-carrying pigs in enclosed and often poorly ventilated spaces—and the export of selectively bred pigs from Europe and United States to different continents [153]. Following the transcontinental exportation of zoonotic *S. suis* strains, new epidemic lineages appear to have emerged in Asia, where reports of human and porcine infections remain prevalent [154]. Cases of community-acquired *S. suis* infections among humans with no direct contact with pigs are becoming increasingly common [155,156,157,158]. Furthermore, cases of *S. suis* infections and *S. suis* isolates have been recently identified in cattle, lamb, dogs and other animals [159,160,161,162].

### 4.1. Phages of S. suis

Data from studies that have characterised phages that infect *S. suis* are limited. To date, only five lytic phages (SMP [163] and SS1, SS2, SS3, SS4 [164]) have been isolated against the pathogen. SMP is the first and only isolated virulent phage of *S. suis* with associated sequence data. Therefore, several studies have been conducted to characterise the phage at in vitro and genomic levels. The SMP phage was determined to have a narrow host range, as it could only infect 2 out of 24 serotype 2 strains included in the screening [163]. Other studies have chemically induced temperate phages from *S. suis* isolates [165,166,167]. In addition, *S. suis* genomic studies have contributed to the few available *S. suis* phage sequences [165,167,168,169]. However, the effectiveness of phages in preclinical animal models of *S. suis* infections has yet to be reported.

Understanding host–phage interactions is key in the evaluation of phages as therapeutic agents against specific bacteria. These interactions include the preservation of phage populations through integration (prophage) in host genomes and the defence mounted by host bacteria via the expression of anti-phage systems. To shed light on the *S. suis* phage landscape, we analysed 133 publicly available whole genome sequences of *S. suis* for the presence of prophages and anti-phage defence systems. The sequences/strains (supplementary data Table S1) were randomly selected, and antiviral defence systems were identified using the web tool PADLOC (https://padloc.otago.ac.nz/padloc/) (accessed on 22 June 2022) [170]. Detection of candidate prophage regions was performed using PHASTER [171]. The predicted regions were categorised (scored) into full-length prophage (>90), putative full-length prophage (90–70) or incomplete prophage (<70) based on the number of coding sequences (CDS) and phage-related genes. To verify predicted regions, intact prophage sequences ≥10 kb were annotated using RAST [172]. A phage proteomic tree was generated using ViPtree [173] and visualised in iTOL.

**Figure 2:**
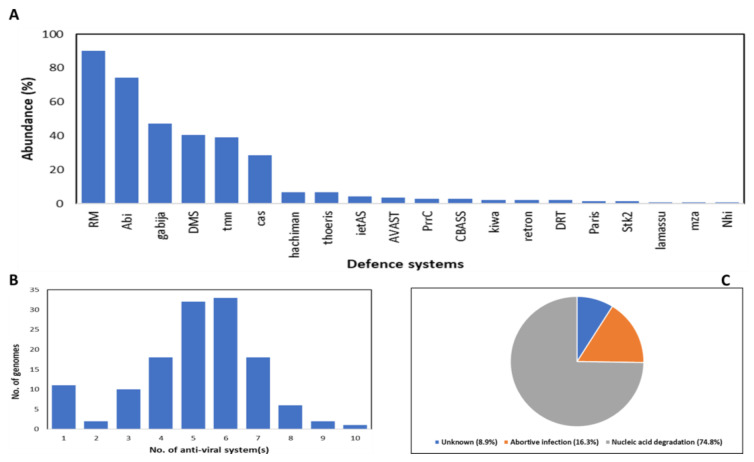
Analysis of anti-viral defence systems detected among 133 publicly available *S. suis* genomes using PADLOC. (**A**) Abundance of defence systems calculated as the percentage of genomes (out of the total 133) that encode a specific defence system. (**B**) Total number of anti-viral systems per genome. (**C**) Categories of anti-viral systems based on molecular mechanism.

The 133 *S. suis* genomes encoded a total of 1890 anti-viral proteins representing 20 distinct anti-viral systems (33, including system subtypes, Supplementary data Table S2). All strains encoded at least one defence system and a maximum of up to 10 systems in a single genome. The average number of systems per genome was calculated as 5.1, with 69.2% of strains encoding five or more systems. Restriction-modification (RM) systems (particularly RM type I) are the most abundant, nearly ubiquitous (90.2%) antiviral defence system family in the *S. suis* genomes. This was higher than the overall abundance (83%) of RM systems in most prokaryotic genomes [174]. Abortive infection systems, including AbiD, AbiE, AbiG, AbiO, AbiQ and AbiZ, were present in 74.4% of the analysed genomes, followed by the less-characterised tmn system (39.1%). Intriguingly, CRISPR-Cas systems (type I and II) were detected in only 28.6% of the genomes. Other recently identified systems such as Hachiman, Thoeris, AVAST and Stk2 were each only identified in less than 7% of the genomes. The different anti-viral systems identified in *S. suis* genomes could be grouped into three categories: DNA/RNA degradation, abortive infection, and systems of unknown mechanisms (Figure 2C). The innate RM and adaptive CRISPR-Cas immune systems target and degrade viral nucleic acids while the signalling systems CBASS, Stk2, Paris, and PrrC, are involved in “cell suicide”. Altogether, of the 1890 predicted proteins, 74.8% were functionally associated with degradation of viral nucleic acids.

### 4.2. High Prophage Prevalence and Diversity in S. suis Genomes

**Figure 3:**
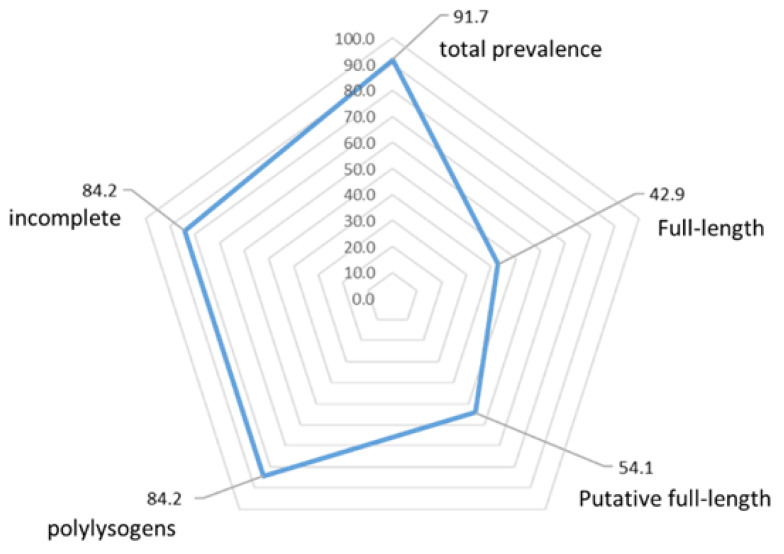
Prevalence of prophages in *S. suis* genomes. Predicted regions were categorised into full-length (42.9%), putative full-length (54.1%), and incomplete prophages (84.2%). Of the 133 genomes, 84.2% are polylysogens—strains harbouring more than one phage region.

The 133 genome sequences of *S. suis* isolated from both diseased and healthy pigs or humans from 11 countries were queried for the presence of prophage sequences. Of the analysed genomes, 91.7% harboured at least one prophage (Figure 3). In total, 501 phage regions were identified, which included 330 incomplete regions, 100 putative full-length prophages, and 71 full-length prophages (Supplementary data Table S3). The number of prophage sequences in prophage-carrying genomes ranged from 1 to 10, with an average of 3.8 prophages per genome. Full-length prophages were detected in 42.9% of the genomes and carried essential phage genes representing a reservoir of genetic material for the development of *S. suis* therapeutic phages or derived enzymes. The average size of the full-length prophages is 45.5 kb, which is larger than the 36.0 kb of the *S. suis* SMP phage. Only full-length prophages were included in further analyses.

**Figure 4:**
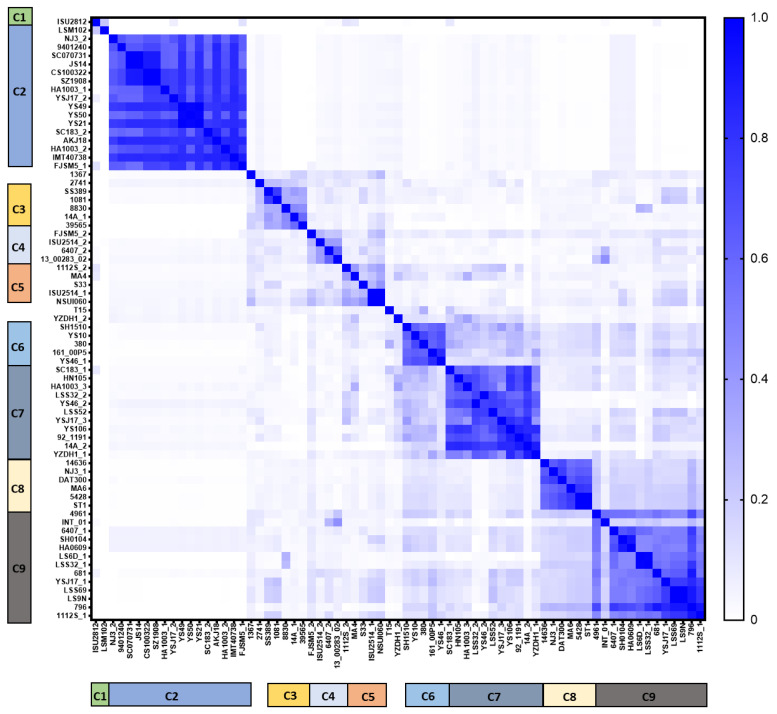
Whole proteomic heatmap of the 71 full-length *S. suis* prophages. Nine clusters (C1–C9) and four singletons were identified. Members within each cluster share ≥ 50% (≥0.5) proteomic identity. Scale bar on the right represents sequence identity. Blue (1.0) represents highest identity, and white (0) represents no proteomic pairwise identity.

**Figure 5:**
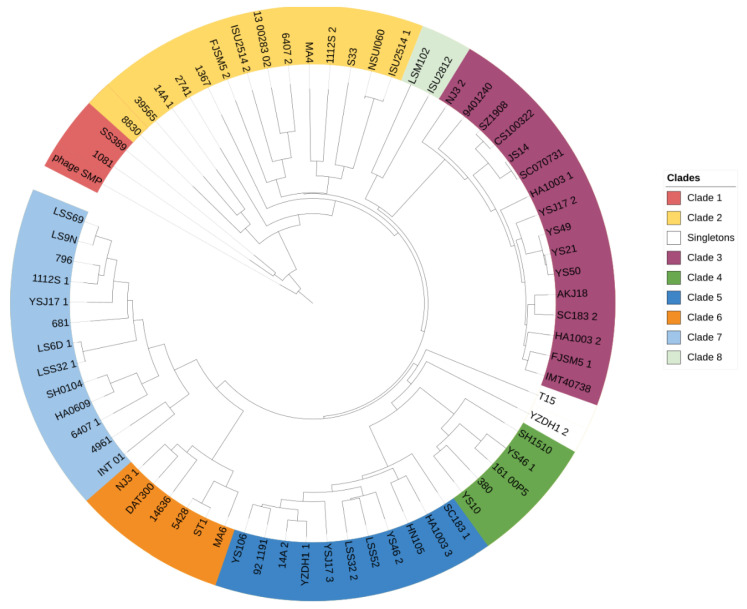
Phylogenetic tree of *S. suis* (pro)phages. Tree was constructed from DNA sequence of the 71 full-length prophages with phage SMP as root. Computations of distance matrix and proteomic tree generation were performed in ViPtree. The final tree was visualised using iTOL. Clades are shown in different colours.

The DNA sequences of all full-length prophages were aligned, and gene similarity and prediction was performed. Although the pairwise identity ranged from 0–100%, about 72.4% of prophage-against-prophage identities were less than 10% (13.5% overall average similarity, Supplementary Materials Table S4). Nonetheless, a proteomic heatmap revealed nine clusters (identity ≥ 50%) and four singletons (Figure 4). Similarly, a phylogenetic tree using phage SMP as the root showed a similar clustering pattern (Figure 5). In the case of strains harbouring more than one full-length prophage, there was a wide distribution of the prophages among clades, suggesting low similarity and high diversity (Figure 5). Prophages within a clade were retrieved from hosts isolated from different locations and diverse serotypes, which suggests wide prevalence among *S. suis* serotypes and geographical regions.

### 4.3. Protein-Encoded S. suis Prophages

**Figure 6:**
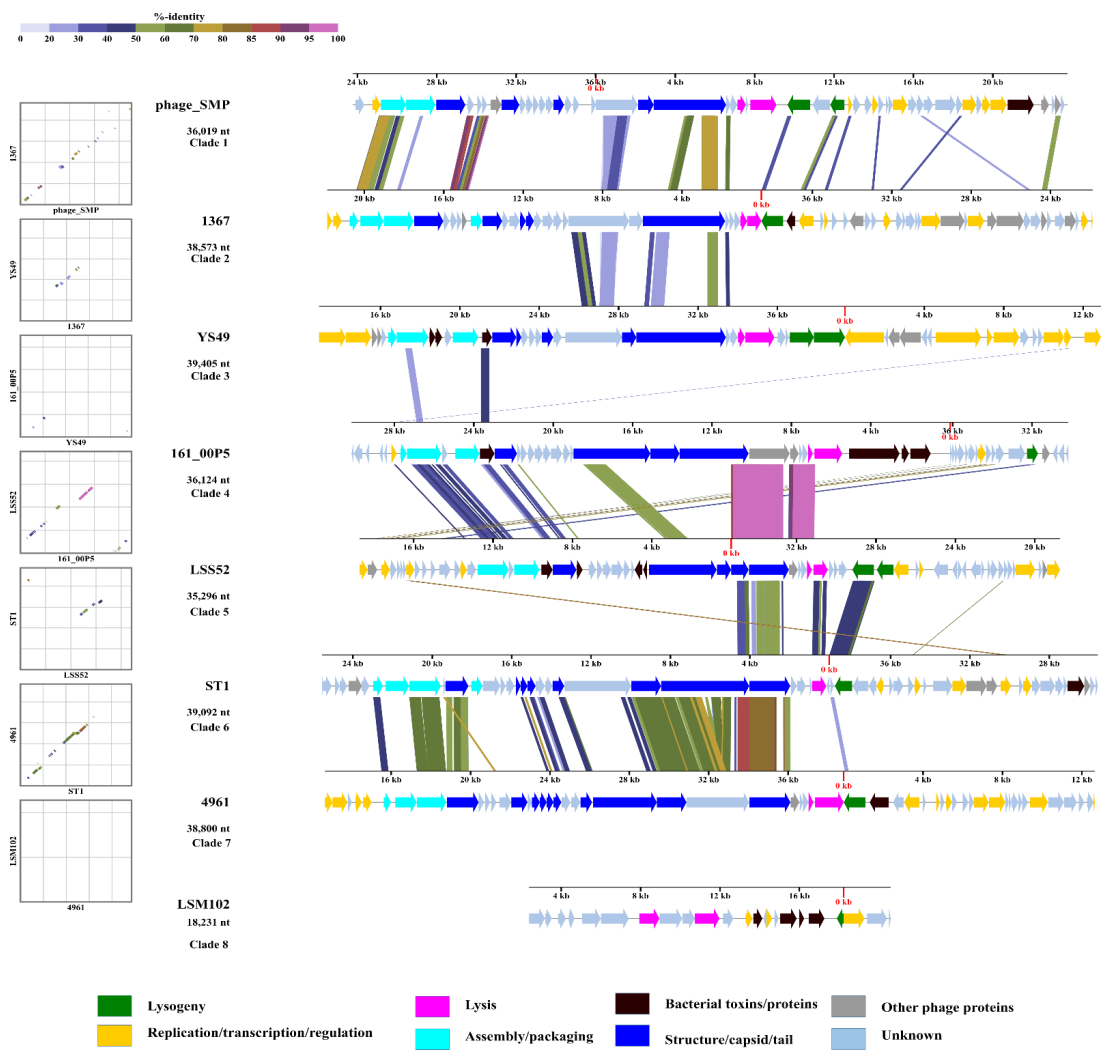
Schematic representation of (pro)phage CDS similarity and organisation. Representative full-length (pro)phage genomes from each clade (of similar genome size) were selected and aligned. Horizontal colour-coded arrows indicate gene function (key on the bottom). Coloured diagonal and vertical lines (alignment) represent percent identity of genes. Dot plot of pairwise alignment indicated on the far left. Colour scale for alignments and dot plots presented in the top left.

Full-length prophages encoded an average of 64 CDS (coding sequence) per sequence (ranged 13–141 CDS), among which, an average of 49.7% could be assigned a putative function. Bacterial proteins associated with virulence, such as toxin/antitoxin, efflux pumps, zeta toxin, and virulence-associated protein E, were detected in the prophages. In addition, anti-phage defence genes such as RMs were detected in prophage sequences, indicating that some bacterial defence systems are prophage-encoded (Supplementary Materials Table S5). The functional analysis revealed that most of the predicted genes are associated with phage morphogenesis, replication and transcription, lysis, and lysogeny. Representative prophages (8), one from each clade, were aligned. A modular arrangement of genes into various phage functions was observed (Figure 6). The majority of prophage genes were transcribed in the same direction, except the lysogeny modules, which were generally transcribed in the opposite direction. Although alignment scores were very low among different clusters, members within the same cluster showed high gene similarities (Supplementary Materials Figure S1).

### 4.4. Relationship between Bacterial Genome Size, Number of Anti-Viral Defence, and Prevalence of Prophages

**Figure 7:**
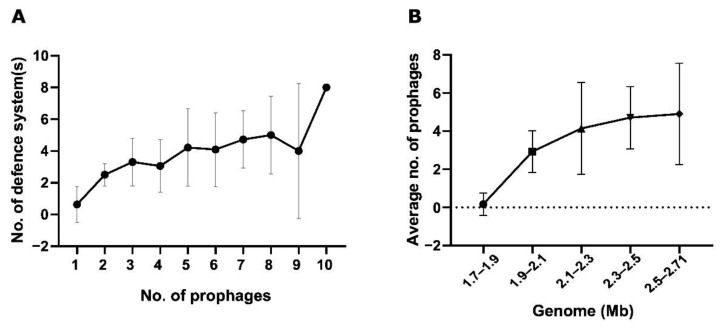
Association between number of prophages and defence systems or host genome size. (**A**) Average number of defence systems correlated with number of prophages identified in a genome (Spearman’s r = 0.40, *p* < 0.0001). (**B**) Average number of prophages per host genome size (Spearman’s r = 0.56, *p* < 0.0001).

The number of prophages in a genome was correlated with the total anti-viral systems identified per genome (Figure 7A). There is a moderate positive correlation between the two parameters (Spearman r = 0.40, *p* < 0.0001). The influence of host genome size on the association between prevalence of prophages and number of defence systems was controlled using a correlation matrix (*p* < 0.0001). Similarly, a moderately positive correlation was observed between the host genome length and the number of prophages (Spearman’s r = 0.56, *p* < 0.0001). Up until 2.5 Mb, the number of prophages increased with increasing genome length (Figure 7B).

## 5. Implications for the Development of Therapeutics

Persistent lysogeny and anti-viral mechanisms are critical phage–host interactions that affect the success of phage infection, particularly in the application of phages as therapeutic agents. We investigated the prevalence of prophages in *S. suis* and the anti-phage defence systems encoded in the bacterium’s genome. All strains encoded at least one defence system, with RM systems being the most abundant. The high abundance of RM systems is consistent with a previous study of Type I RM systems in *S. suis,* which identified 95% of isolates encoding the defence system [175]. Interestingly, CRISPR-Cas systems were detected in a small fraction of *S. suis* genomes as opposed to the overall estimated abundance in bacteria (~50%) [176]. Moreover, the opposite is the case among other streptococci, such as *S. thermophilus,* in which all strains possibly encode the system, or in *S. mutans,* where 95% encode at least one type of CRISPR-Cas [177]. Recent studies have unearthed previously unknown anti-viral systems within bacterial defence islands. This suggests that current quantification of defence systems underestimates the prokaryotic defence arsenal. Except for tmn (39.1%) and gabija (21.1%), each of these newly described systems were present in less than 7% of *S. suis*. We categorised the defence systems on the basis of their molecular mechanism. Nucleic acid degradation systems were the most common mechanism in the *S. suis* defence arsenal. This is in line with the estimation for prokaryotes [174].

We identified 501 prophage regions, averaging 3.8 prophages per genome. The prevalence in *S. suis* is higher than has been reported for other Streptococcal species such as *S. pneumoniae* (1.4 per genome) [178] and *S. mutans* (1.5 per genome) [179]. Of the 501 prophages, 71 full-length prophages encoding essential phage genes were further analysed. It is possible that the number of full-length prophages was underestimated when using PHASTER because the majority (60.2%) of the genomes in this study were retrieved as multiple contigs. Thus, there is a possibility of prophages encoded on separate contigs, in which case they would be predicted to be incomplete. More than half of the intact prophages were detected in the genomes that were complete *S. suis* chromosomes. However, this observation could also be a function of the sample size. An all-against-all genomic similarity revealed little proteomic relatedness among full-length prophages suggesting a high genetic diversity. Two patterns can be described for phage groups with low gene relatedness. Firstly, along the genomes of phages of common ancestry, several gene homologs of little similarity exist. Alternatively, phage mosaicism may arise from genetic transfer between dissimilar phages such that only a small fraction of the genome would contain highly similar homologs. The latter trend is consistent with the low inter-cluster relatedness as we qualitatively show in a schematic representation of functional genome alignment (Figure 6). Furthermore, it has been demonstrated that temperate phages and virulent phages, employ high and low gene flux, or only low gene flux evolutionary modes, respectively. Particularly in *Synechoccocus* phages among which low gene flux is favoured, there is an observed clusters of genetically-distinct populations and constrained mosaicism [180]. Future investigations may explore the rates of genetic transfer and its influence on the diversity, host specificity and host range of *S. suis* temperate phages. Despite the low genetic similarity, all representative prophages from the clusters displayed a modular architecture such that essential phage genes could be categorised into structural, lysis and lysogeny, replication/transcription/metabolism, and packaging. Thus, it is likely that these temperate phages can be induced in vitro and explored for their potential for therapeutic applications.

The role of prokaryotic defences such as CRISPR-Cas in preventing successful phage infection has been described by others [181,182]. We hypothesised that the abundance and diversity of anti-viral defence systems would have a negative effect on the prevalence of prophages in a genome. On the contrary, we found a positive correlation between the number of anti-viral systems encoded in a genome and the number of prophages it harboured. We also found a similar association between host genome size and the prevalence of prophages. Strains with small genomes harboured no or fewer prophages compared to strains with larger genomes. This raises the questions of why and how a trifaceted association of *S. suis* host genome size, the prevalence of prophages, and the abundance of defence systems exists. In *Salmonella* and *E. coli,* larger genomes were associated with more integration hotspots. In particular, there seemed to be a selection for integration sites within intergenic and regions of low gene expression rather than highly transcribed regions. In highly transcribed regions, spillovers from transcribed genes could trigger phage gene expression, which can reduce host abundance and consequently the temperate phage population following a switch to lytic cycle [183]. Future investigations could examine integration sites utilised by temperate phages infecting *S. suis* and how this influences prophage prevalence among different strains. Following integration, temperate phages can confer fitness to the host. For instance, in the context of the correlation between defence systems and the number of prophages, annotations revealed the presence of phage-encoded RM systems (Supplementary Materials Table S5). Thus, selection for such traits could lead to saturation of host chromosomes by prophages that encode fitness genes (including virulence and AMR), which will influence the diversity of the anti-viral arsenal of the host.

It has been suggested that the phage population in the gut microbiota is sustained predominantly through lysogenic replication, with spontaneous inductions rather than repetitive lytic cycles or frequent triggered prophage inductions [184]. In the lysogenic state, the prophage exerts superinfection immunity, which acts to prevent subsequent phage infections as demonstrated in mycobacterial phages [185]. We have shown that there is a high prevalence of prophages in the genomes of the bacterium; however, only five lytic phages against *S. suis* have been reported to date. It is possible that in this species, there is an evolutionary preference for lysogeny in the nasopharyngeal microbiota of the animal hosts. Notwithstanding, the prophages identified serve as building blocks for the development of candidate phages for therapeutic application in *S. suis* infections.

### Future Perspectives

A potential approach to explore untapped (pro)phages against *S. suis* and other bacterial pathogens is through genetic engineering. Current genetic manipulation techniques such as recombineering, yeast artificial chromosome (YAC), CRISPR-Cas systems, and in vitro synthetic genome assembly have been leveraged to custom-design phages with desired characteristics [186]. Modifications through deletions and insertions of targeted genes or gene clusters can be employed to broaden host range of a phage, increase phage efficacy through evasion of specific host defences, and improve the safety of phages. By using a YAC platform, Ando et al. constructed synthetic T3 and T7 phages with swapped gp17—a host determinant. The resulting hybrids successfully cross-infected hosts of both phages. Using the same technology, engineered coliphage T7 could selectively eliminate *Klebsiella* sp. and *Yersinia* sp. in a mixed population [187]. Alternatively, the CRISPR-Cas system of *S. thermophilus* has been used to introduce specific point mutations, large deletions, and ORF replacement, with relatively high efficiencies. This model was adapted for the construction of escape phages through the introduction of a methyltransferase gene such that the resulting phage mutants evaded the R/M system of the host. This modification significantly improved the bacteriolytic efficiency of the *S. thermophilus* phage 2972 [188]. A synthetic genome assembly has been used to produce infectious viral particles from short phage DNA fragments [189], allowing for the deletion of lysogeny-associated genes and acquired virulence genes within phage genomes. Obligately lytic phages have been constructed from temperate phages for the control of *Mycobacterium abscessus* in cystic fibrosis patients [57,58]. Similar approaches can be explored in the custom design of lytic phages against *S. suis* using their prophages as building blocks.

Moreover, co-evolution “phage training” experiments have been adopted to adapt phages to oppose host defences and widen the host range [190]. The concept has been used to pre-adapt phages for treatment in a human *K. pneumoniae* infection [53]. However, this coevolutionary dynamic is yet to be validated in vivo for other clinically relevant species. Lastly, “Enzybiotics” explores the use of phage-encoded enzymes in the control of bacteria. Phage-derived enzymes and endolysins targeting *S. suis* have demonstrated flexibility in engineering, low toxicity profiles, and broad efficacy against the pathogen and other streptococcal species, which support their potential for translational development [191,191,192,193,194].

Although naturally occurring phages possess a green appeal, the incorporation of genetically modified organisms (GMOs) in food may interfere with their approval by regulatory bodies. To date, the application of genetically modified phages in the treatment of infections in humans have been limited to rare cases with specific therapeutic circumstances meriting exemptions for their usage. Any additional barriers to approval will lessen commercialisation. Similarly, consumer reticence towards GMOs will decrease the likelihood of their application. Compelling research will be needed to prove the efficacy, safety and scalability of genetically engineered phages required by authorising bodies. Researchers also have a duty to engage with the public and policy groups to impress upon them the importance of phage solutions to tackle the catastrophe of antimicrobial resistance. As the prevalence of the problem continues to grow, this consumer reticence towards GMOs may become less of a challenge.

## 6. Conclusions

The emergence of antibiotic-resistant zoonotic bacteria poses a threat that transcends the food industry, affecting the overall health of the environment and other animal populations. The efficacy and safety of phages have been demonstrated in several bacterial infections, with some commercial products already approved in several countries for use in food production. However, there are other zoonotic bacteria, including *S. suis*, for which phage applications remain underexplored. We provide insights on the *S. suis* phage landscape and also paint a quantitative picture of the anti-phage arsenal of *S. suis*. Our findings reveal the diversity and abundance of *S. suis* prophages, which can be used as building blocks for synthesising safe lytic phages for application in food and medicine.

## Figures and Tables

**Table 1:** Advantages and limitations associated with phages as antibacterial agents.

Feature	Advantages	Limitations/Remarks
Specificity	Targets bacterial strains in a highly specific manner, thereby targeting only the intended bacteria and leaving bystander members of the resident microbiota unharmed	Strain specificity typically results in narrow target range compared to antibiotics. A mixture of several phages (cocktail) may be required for optimal bacteria clearance
Mode of action	Autodosing: ability to replicate at the site of infection and lyse bacterial cells	Temperate phages may integrate into bacterial chromosomes (prophages) and be passively replicated without resulting in bacterial lysis
Toxicity, safety profile and immunogenicity	Phages do not infect or have serious adverse effect on eukaryotic cells. Endotoxins can be easily removed during phage purification.	Efficacy of phages may be reduced through neutralisation by animal immune system
Efficacy against MDR	Effective against multidrug resistant bacteria	Some phages may encode antibiotic resistance to genes and toxins that confer extra fitness to bacteria
Resistance	Specificity of phages limits the widespread use of specific phages in different infections, thus reducing the chances of resistance development by bacteria	Bacteria encode anti-phage systems such as abortive infection, restriction-modification, gabija, CRISPR-Cas, DISARM, etc., that interfere with successful phage infection
Production	Natural; can be isolated from diverse clinical and environmental sources and characterised rapidly compared to antibiotic discovery and development	Difficulty in isolating good therapeutic phage candidates against specific species or strains such as *S. suis* Regulatory approval for the use of phages as therapeutics is onerous and has limited the commercialisation of phage products
Administration	Can be incorporated into feed or water and administered to animals	Challenges in formulation and stabilisation of phage preparation for therapy have been reported

**Table 2:** Some applications of phages in human infections (2017–2022).

Condition/Infection	Phage Intervention	Remarks	Reference
Cystic fibrosis/*Achromobacter xylosoxidans*	Phage cocktail (3 × 10^8^ pfu/mL) for 20 d via inh, p.o. Treatment was repeated 4 times after initial PT at 1, 3, 6 and 12 mo.	Dyspnea resolved and cough reduced Increased lung function	[49]
Crohn’s disease/MDR *Klebsiella pneumoniae*	3 week cycle single phage treatment 10^6^ pfu/mL p.o., 10^6^ pfu/mL rectal	15 days after first PT treatment, no MDR *K. pneumoniae* was isolated from patient’s stools, rectal swabs, urine and the ureteral stents	[50]
Necrotising pancreatitis/systemic *Acinetobacter baumannii*	3 different phage cocktails (1 × 10^9^ pfu/mL i.c. for 18 weeks and 5 × 10^9^ pfu/mL i.v. for 2 or 16 weeks)	Patient awoke from coma; mental health and renal function improvedPatient was discharged on day 245	[51]
Aorto-cutaneous fistula/*Pseudomonas aeruginosa*	Single i.o. dose of 10^8^ pfu/mL phage OMKO1	Blood cultures tested negative for *P. aeruginosa* after for 4 weeks	[52]
Fracture-related infection (FRI)/*Klebsiella pneumoniae*	100 mL of 10^8^ pfu/mL on day 1 and 10^7^ pfu/mL was instilled on surgical wound via catheter t.i.d. up to day 5	Improved microbiological, radiological and blood parameters 3 months post-phage therapy FRI was controlled	[53]
Disseminated cutaneous/*Mycobacterium chelonae* infection	i.v. of 10^9^ pfu/mL b.i.d. for > 6 months	Discharged on day 4 following no adverse effects and improved laboratory markers No evidence of granulomas 2 months after beginning of phage therapy	[54]
Chronic vascular graft infection/*Staphylococcus aureus*	local application of 20 mL 10^9^ pfu/mL via drainage q.12 h for two days	Negative blood culture after last day of phage treatmentNo sign of graft infection	[55]
Sternal wound abscesses/*P. aeruginosa*	Single 4 ml 4 × 10^10^ pfu/mL i.o.	Wound was completely healed *P. aeruginosa* was undetectable post-phage therapy	[55]
Cystic fibrosis/*P. aeruginosa*	8 weeks of 4 × 10^9^ pfu/mL i.v., q.6 h	Resolution of renal function, white blood cell counts and fever No CF exacerbation or recurrence of *P. aeruginosa* 100 days post-PT	[56]
Cystic fibrosis/*Mycobacterium abscessus*	Single topical 10^9^ pfu/mL cocktail on wound 10^9^ pfu/mL i.v., q.12 h for 32 weeks	Negative serum and sputum cultures Positive skin nodule swabs up to 5 months post-PTNo sera phage neutralisation	[57]
Lung disease/*M. abscessus*	Up to 10^9^ pfu/mL i.v., b.i.d. for > 6 months	*M. abscessus* cultures positive (6 of 7) through day 96 Most recent cultures (days 116–362) were negative (90%) Patient successfully underwent lung transplant post-PT	[58]

Abbreviations: d: days; inh: inhalation; p.o.: orally; i.c.: intracutaneous; mo: months; i.v.: intravenous; i.o.: intraoperative; t.i.d.: three times a day; b.i.d.: twice a day; q.12 h: every twelve hours; q.6 h: every six hours; pfu: plaque forming unit.

**Table 3:** Summary of phage studies in food matrices and surfaces.

Food Matrix/Surface	Target Pathogen	Phage Treatment	Results/Log Unit Pathogen Reduction
Celery [116] Enoki mushrooms	*L. monocytogenes*	cocktail	Reduced initial 5.0 by 2.2 (celery) and 1.8 (mushroom)
Salmon meat [117] Orange juice	*L. monocytogenes*	SH3-3 phage	Target undetectable at 72 h compared to control (2.31)
Milk [118] Chicken	*S.* Typhimurium	cocktail	Milk: undetectable by 2 h (MOI 1000) or 12 h (MOI 100) at 25 °C Chicken: undetectable by 2 h (MOI 1000) or 6 h (MOI 100) at 25 °C
Milk [119] Apple juice	*Salmonella*	phage LPSTLL	Initial 3.0 reduced by 2.8 in milk and by 0.52 in apple juice
Chicken-lettuce salad [120]	*S.* Enteritidis	SapYZU01	Initial 5.1 reduced by 3.4
Meat and vegetables [121]	*E. coli*	Phage DW-EC	Initial 6.0 reduced by 43.38–87.89% on the foods
Mung beans [122]	*E. coli*	phage Sa45lw	Initial 4.8 reduced by 2.0 within 6 h
Chicken [123] Mutton	*C. jejuni*	phage CJ01	Initial 4.0 reduced by 1.68 in chicken and 1.70 in mutton
Acid curd [124] Rennet curd	*S. aureus*	cocktail	undetectable by 4 h in acid curd or 1 h in rennet curd
Chicken breast [125]	*Shigella Flexneri*	phage SflS-ISF001	Initial 4.0 reduced beyond 2.0
Stainless steel [126]	*L. monocytogenes*	cocktail	Initial ~5.4 was undetectable by 75 min
Rubber [127] polyethylene Stainless steel (SS)	*Cronobacter sakazakii*	phage JK004	inhibition rate for 6 h for rubber, polyethylene or SS was 99.95, 99.83, or 99.84%, respectively
Stainless steel [128]	*S.* Enteriditis and Typhimurium	phages BP1369 and BP1370	Undetectable after 144 h at 10 °C
Stainless steel [129]	*E. coli*	phage AZO145A	Initially 4.8 reduced by 2.9 in 24 h

**Table 4:** Some commercially available phage products for food industry applications.

	Product Name	Target Pathogen	Application
Proteon Pharmaceuticals (Poland)	Bafasal^®^	*Salmonella* spp.	Feed or water additive
	Bafador^®^	*Aeromonas hydrophila* and *Pseudomonas fluorescens*	Feed additive for aquaculture
Intralytix (USA)	INT-401™	*Clostridium perfringens*	In-feed or water additive
	PLSV-1™	*Salmonella* spp.	Animal health care
	Ecolicide PX™	*E. coli O157:H7*	Hides of live animals
	ListShield™	*L. monocytogenes*	Food and surface decontamination
	ShigaShield™	*Shigella* spp.	Decontamination of ready-to-eat (RTE) foods
	SalmoLyse^®^	*Salmonella* spp.	Decontamination of pet food
ACD Pharma (Norway)	CUSTUS^®^ YRS	*Yersinia*	Treatment of fish environment in aquaculture
Phagelux (China)	LUZON	*Staphylococcus*, *E. coli*, *P. aeruginosa* or *Salmonella*	Control of infections in pig farms
	SHIJUNSHA	*Staphylococcus*, *E. coli*, *P. aeruginosa* or *Salmonella*	Control of infections in poultry farms
OmniLytics Inc. (USA)	Agriphage™	*Xanthomonas campestris*, *Pseudomonas syringae*	Infection control on pepper and tomato
CJ Cheiljedang Corp. (South Korea)	Biotector	*Salmonella*, *C. perfringens* and *E. coli*	Disease management on farms
Micreos Food Safety (The Netherlands)	PhageGuard Listex™	*L. monocytogenes*	Decontamination of RTE and frozen foods
	PhageGuard E	*E. coli*	Decontamination of food products
	PhageGuard S	*Salmonella* spp.	Decontamination of food products

## Data Availability

All *S. suis* genome data was downloaded from NCBI https://www.ncbi.nlm.nih.gov/nuccore/?term=streptococcus+suis (accessed on 14 October 2021). Information on genomes used during analysis, including accession numbers, can be found in the Table S1_*Streptococcus suis* strain_genome information.

## Supplementary Materials

The following supporting information can be downloaded at: https://www.mdpi.com/article/10.3390/v14091996/s1, Figure S1: Schematic representation of prophage CDS similarity and organisation; Table S1: *Streptococcus suis* strain/genome information; Table S2: Predicted anti-viral defence systems (position, HMM name and accession, protein name); Table S3: Summary all detected prophage regions (length, GC %, number of CDS); Table S4: Prophage annotations sheet; Table S5: Pairwise protein identities (%) of full-length prophages.
