# Supplementary material for: From Farm to Fork: Streptococcus suis as a Model for the Development of Novel Phage-Based Biocontrol Agents
Source: Viruses. 2022 Sep 9;14(9):1996. doi: 10.3390/v14091996 (PMC9501460; doi:10.3390/v14091996)
Supplement: Supplementary file 1 [file viruses-14-01996-s001.zip › Figure S1_Schematic representation of prophage CDS similarity and organisation.pdf]

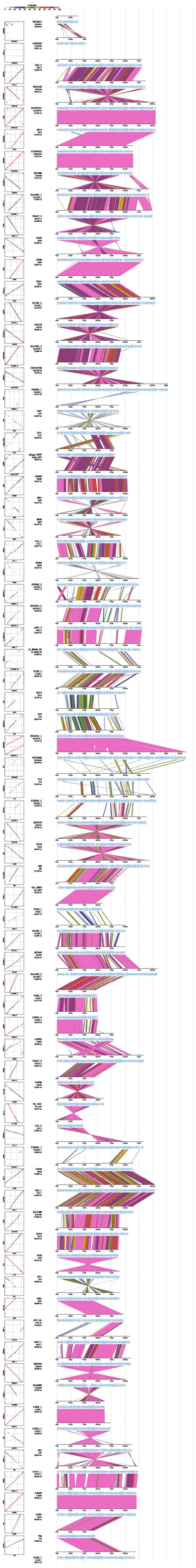

Supplementary figure S1. Schematic representation of prophage CDS similarity and organisation. All 71 full-length prophage sequence retrieved from *S. suis* genomes were aligned. Horizontal arrows indicate individual CDS. Coloured diagonal and vertical lines (alignment) represent percent identity of genes. Dot plot of pairwise alignment indicated on the far left. Colour scale for alignments and dot plots presented in the top left.
