# Supplementary figures and images for: From Farm to Fork: Streptococcus suis as a Model for the Development of Novel Phage-Based Biocontrol Agents
Source: Viruses. 2022 Sep 9;14(9):1996. doi: 10.3390/v14091996 (PMC9501460; doi:10.3390/v14091996)

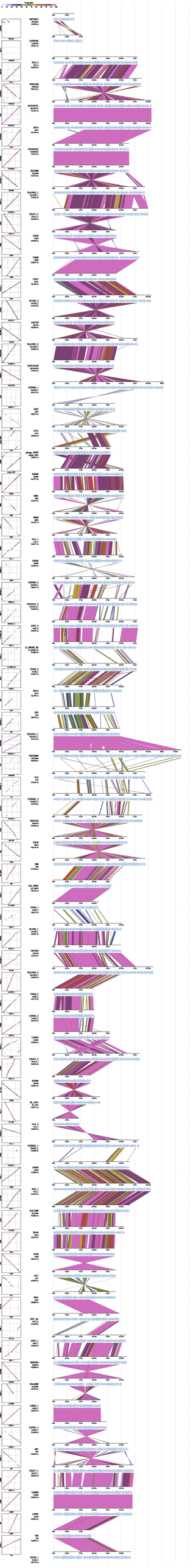

Supplement: Supplementary file 1 [file viruses-14-01996-s001.zip › Figure S1_Schematic representation of prophage CDS similarity and organisation.jpg]
